# The decreased expression of *IKBKE* in systemic lupus erythematosus

**DOI:** 10.1007/s10067-020-05006-6

**Published:** 2020-03-07

**Authors:** Tingting Zhu, Jiaqi Hong, Zongwen Shuai, Shengqian Xu, Danfeng Qian, Xiaojie Hong, Yaoguang Liu, Min Chen, Ziyuan Meng, Lijun Zheng, Danlin Zheng, Xuejun Zhang, Lu Liu

**Affiliations:** 1 Institute of Dermatology and Department of Dermatology, the First Affiliated Hospital, Anhui Medical University, 81 Meishan Road, Shushan District, Hefei, 230032 Anhui China; 2 Department of Rheumatology, The First Affiliated Hospital of Anhui Medical University, Hefei, Anhui China; 3 Department of Medical and Molecular Genetics, King’s College London, London, UK

**Keywords:** *IKBKE*, NF-κB signaling pathway, Single nucleotide polymorphism, Systemic lupus erythematosus

## Abstract

**Objective:**

The *IKBKE* has been proven to be associated with systemic lupus erythematosus (SLE) in a genome-wide association study (GWAS) conducted by our group. The objective of the recent study is to investigate the contribution of *IKBKE* functional variants (rs2297550) to SLE.

**Methods:**

We detected the regulatory effect of rs2297550 on *IKBKE* expression by expression quantitative trait loci (eQTL) study. Then, we investigated the differences of *IKBKE* mRNA expression levels in peripheral blood mononuclear cells (PBMCs) between 135 SLE patients and 130 healthy controls using quantitative real-time PCR (qRT-PCR). We further analyzed the association of SLE clinical characteristics with *IKBKE* mRNA expression and rs2297550 polymorphisms.

**Results:**

The results of eQTL indicated the genotype “GG” of single-nucleotide polymorphism (SNP) rs2297550 was associated with lower expression levels of *IKBKE* (*P* = 0.022) in normal controls. Compared with the healthy control group, the expression levels of *IKBKE* mRNA in patients with SLE were significantly decreased (*P =* 2.32 × 10^−12^). In clinical characteristics, we found that *IKBKE* mRNA expression levels were associated with vasculitis (*P* = 0.015) and increased C-reactive protein (CRP) (*P =* 0.021) in SLE patients.

**Conclusion:**

In this study, we not only detected that the variant rs2297550 of *IKBKE* may be closely related to SLE, but also proposed functional hypotheses for the association signals.

**Table:** 

**Key Points** • *The rs2297550 is located in a region with transcriptional regulatory function and may regulate the expression of IKBKE* via *these regulatory elements.* *• The genotype “GG” of SNP rs2297550 was associated with lower expression levels of IKBKE.* *• The expression of IKBKE mRNA was decreased in SLE patients compared with healthy controls.* *• IKBKE contributes to the clinical characteristics of SLE.*

**Electronic supplementary material:**

The online version of this article (10.1007/s10067-020-05006-6) contains supplementary material, which is available to authorized users.

## Introduction

Systemic lupus erythematosus (SLE) (OMIM 152700) is a systemic autoimmune disease with various complicated clinical characteristics. Over the past several decades, genome-wide association studies (GWASs) have recognized more than 100 single nucleotide polymorphisms (SNPs) closely related to SLE [1–3]. Our group firstly detected SNP rs2297550 of *IKBKE* was associated with SLE in a trans-ethnicity GWAS between Han Chinese (*P* = 1.52 × 10^−4^) and European (*P* = 1.59 × 10^−4^). We showed the results of SLE susceptible SNP rs2297550 in that study (Supplementary Table 1) [4] and further summarized allelic and genotypic frequencies of rs2297550 in different population according to 1000 Genomes Project Phase 3 (Supplementary Table 2) [5]. *IKBKE* encodes protein IKKε which is an inhibitor of the nuclear factor kappa-B kinase subunit epsilon gene [6]. IKKε belongs to the noncanonical IkB kinase family and has a crucial impact in adjust of the immune response [6, 7]. IKKε is a vital member of activation and transmission of NF-κB signaling pathway [8] which is one of the important factors of the disease pathogenesis of immune system [9]. Here, we analyzed and discovered an additional role of *IKBKE* possibly as a mediator protecting from untoward death of the cell in SLE.

We found the genotype of rs2297550 was associated with *IKBKE* mRNA expression levels and explored the difference of *IKBKE* mRNA expression levels between SLE patients and healthy controls in peripheral blood mononuclear cells (PBMCs). We then revealed the relationship between *IKBKE* mRNA expression and various clinical characteristics of SLE patients. It indicated that *IKBKE* may play a vital role in the SLE mechanism.

## Methods

### Patients and healthy controls

All of 135 SLE patients and 130 healthy controls were enrolled in this study (Table 1); all were enlisted from The First Affiliated Hospital of Anhui Medical University, Hefei, Anhui province, China. The patients of SLE were diagnosed by at least two experienced physicians using the revised American College of Rheumatology SLE classification criteria [10], and their clinical manifestations were documented via a full clinical check-up. The SLEDAI score [11] was performed for each patient according to the clinical findings collected at blood collection time point. The clinical characteristics we defined for each patient were based on laboratory parameters and the medical history recorded by physicians. The clinically confirmed healthy control group had no history of SLE, family history of SLE. All subjects had no other inflammatory process such as rheumatoid arthritis, Sjögren’s syndrome, and so on. Written informed consent was signed by each subject. This study got permission from the Institutional Review Board of the Anhui Medical University of China and was based on the principles of the 1964 Declaration of Helsinki and its later amendments.

**Table 1:** Demographic characteristics of the study population

Characteristic	SLE patients *n* = 121	Healthy controls *n* = 128	*P*
Gender			
Female	117	127	/
male	4	1	/
Age (years) *	37.46 ± 12.86	36.09 ± 9.33	/
BMI	21.33 ± 3.47	21.81 ± 3.28	0.602
Medications			
Corticosteroid use	95(70.37%)	/	/
Antimalarial use	43(31.85%)	/	/
Immunosuppressive use	52(38.52%)	/	/
Genotype			0.823
GG	41 (34.7%)	23 (18.0%)	/ / /
GC	55 (46.6%)	71 (55.5%)	
CC	22 (18.6%)	34 (26.5%)	
Allele			0.945
G	137 (58.1%)	139 (54.3%)	/ /
C	99 (41.9%)	117 (45.7%)	

*The age is when the subjects were enrolled;

BMI: Body Mass Index;

*P* value is considered statistically significant if < 0.05

### Total RNA isolation

We collected blood (5 ml) from all subjects in an anticoagulation tube and then diluted by an equal volume of phosphate buffered saline (PBS). A 15ml centrifuge tube was filled with 5 ml of Ficoll-Paque Premium (GE Healthcare) and 10 ml of the 1:1 diluted blood. PBMCs were extracted by density gradient ultracentrifugation in terms of manufacturer’s requirements. Total RNA was extracted using TRIzol Reagent. In order to obtain high purity RNA, we used the NanoDrop 2000 spectrophotometer to measure samples.

### qRT-PCR analysis

Extracted RNA was reversed transcription (400 ng) with the PrimeScript RT reagent Kit (Takara). The quality of complementary DNA (cDNA) was determined by qRT-PCR. We used glyceraldehyde-3-phosphate dehydrogenase (GAPDH) expression as the internal control. The primers were employed in the qRT-PCR: *IKBKE* primers: forward 5′-TGTCACTGGGGCTGCAGAG-3′ and reverse 5′- GTCGAAGCCCCAGCACTTG-3′. The samples were expanded for 40 cycles on a ViiA 7 Real-Time PCR system (Applied Biosystems) under the following cycles: denatured at 95 °C for 5 seconds, annealed and extended at 60 °C for 30 seconds, finally subjected to dissociation phase. The reactions were performed in a 5 μl reaction system of 10 μM primers. The *IKBKE* mRNA expression levels were calculated using the 2^-ΔΔCt^ method [12].

### Genotyping

Whole blood DNA was extracted using Flexi Gene DNA kits (QIAGEN, Hilden, Germany). The SNP, rs2297550, was genotyped using the Applied Biosystems 7900HT at the State Key Laboratory of Dermatology (Hefei, Anhui, China). According to the manufacturer’s introductions, we genotyped each sample with approximately 20 ng/μl of genomic DNA. The TaqMan SNP Genotyping Assay uses 5 μl reaction volumes, containing 2.5 μl 2 × Master Mix, 0.125 μl 40 × assay consisting of primer and probe, 1 μl genomic DNA and 1.375 μl RNase-Free Water.

### Functional annotation and biological insights

The function and regulatory potential of rs2297550 were assessed by using HaploRegv 4.1 (http://archive.broadinstitute.org/mammals/haploreg/haploreg.php). RegulomeDB database (http://regulome.stanford.edu/) was used to identify DNA features and regulatory elements in the human genome noncoding regions obtaining rs2297550 [13, 14]. GTEx (Genotype-Tissue Expression) Analysis Release V7 (dbGaP Accession phs000424.v7.p2) [15] was used to discover the effect of rs2297550 on *IKBKE* mRNA expression in the skin.

### Statistical analysis

We applied logistic regression to analyze the effects of Body Mass Index (BMI), genotypes and alleles on *IKBKE* mRNA expression. The regulation effect of rs2297550 on *IKBKE* mRNA expression was analyzed by one-way analysis of variance. Because *IKBKE* mRNA expression did not follow the normal distribution, the difference of *IKBKE* mRNA expression levels between SLE patients and healthy controls was analyzed by the nonparametric Mann-Whitney test. The relationship between gene expression levels and clinical characteristics was also investigated by the nonparametric Mann-Whitney test. In addition, to explore whether the clinical characteristics of SLE are related to the different genotypes or alleles of rs2297550, we used the statistical method of χ2 test or one-way analysis of variance. We used SPSS version 24.0 software for all data analysis. Figures were plotted by GraphPad Prism (version 8.01). *P* values (2-tailed) less than 0.05 was considered statistically significant.

## Results

### Association of rs2297550 with *IKBKE* mRNA expression and SLE clinical characteristics

**Fig. 1:**
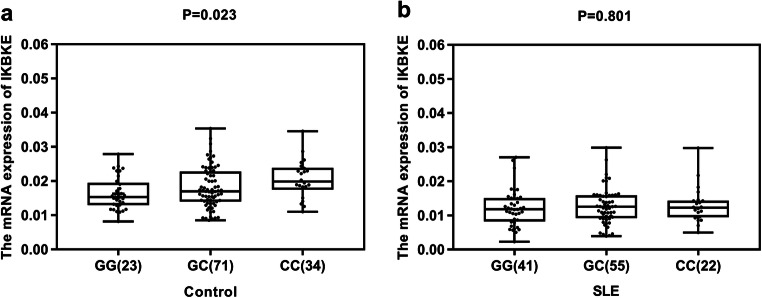
**a** The effect of rs2297550 on *IKBKE* mRNA expression levels in PBMCs from healthy controls. Of the 128 controls, 23 individuals with CC, 71 with GC, and 34 with CC were analyzed. The group with “GG” homozygous has the lowest expression level (*P =* 0.022). **b** The effect of rs2297550 on *IKBKE* mRNA expression levels in PBMCs from SLE patients. Of the 118 patients, 22 individuals with CC, 55 with GC, and 41 with GG were analyzed. The *IKBKE* mRNA expression did not significantly correlate with genotypes of rs2297550 (*P* = 0.801)

We performed eQTL study to detect the effect of rs2297550 on *IKBKE* mRNA expression. The result indicated the genotype “GG” of SNP rs2297550 was associated with lower expression levels of *IKBKE* (*P* = 0.022) in healthy controls (Fig. 1a). However, no significant difference was identified in SLE patients (*P* = 0.801) (Fig. 1b). Additionally, we analyzed the association of some clinical characteristics with different genotype or allele, respectively (Supplementary Table 3). Unfortunately, we did not find that clinical characteristics related to any particular genotype or allele. We considered the limited sample size may affect the statistic power, especially once stratifying SLE patients in clinical features.

### Regulatory effect prediction of rs2297550

The HaploReg v4.1 database predicted that rs2297550 which span the 5′ untranslated region (UTR) of *IKBKE* is a binding site motif. The variant rs2297550 overlapped with gene regulatory elements, i.e., gene promoters and enhancers marked by histone modifications H3K27ac, H3K4me1, and H3K4me3 in some immune cells, kidney, and skin (Table 2). RegulomeDB showed that rs2297550 had transcription factor (TF) binding and DNase sensitivity data types. These results all support the possibility that rs2297550 is located in a region with transcriptional regulation and that the expression of *IKBKE* can be regulated via these regulatory elements. Accordingly, we investigated how rs2297550 affects *IKBKE* expression in GTEx. And the result revealed that rs2297550-G was related to the decreased mRNA expression of *IKBKE* in skin (sun-exposed (*P =* 6.1 × 10^−5^) and not sun exposed (*P =* 9 × 10^−6^)) tissue.

**Table 2:** Functional annotation for rs2297550

SNP	Chr	Gene	Allele	Position (hg19)	Function	Enhancer histone marks	Motifs	Regulome DB score
rs2297550	1	*IKBKE*	G/C	206,643,772	5’-UTR	T cell, B cell, kidney, skin	PU.1_disc3, STAT_disc7, TATA_disc7	4

Allele: minor allele/major allele;

In the Regulome DB score, 4 of score means transcription factor (TF) binding and DNase sensitivity site

### *IKBKE* mRNA expression analysis

**Fig. 2:**
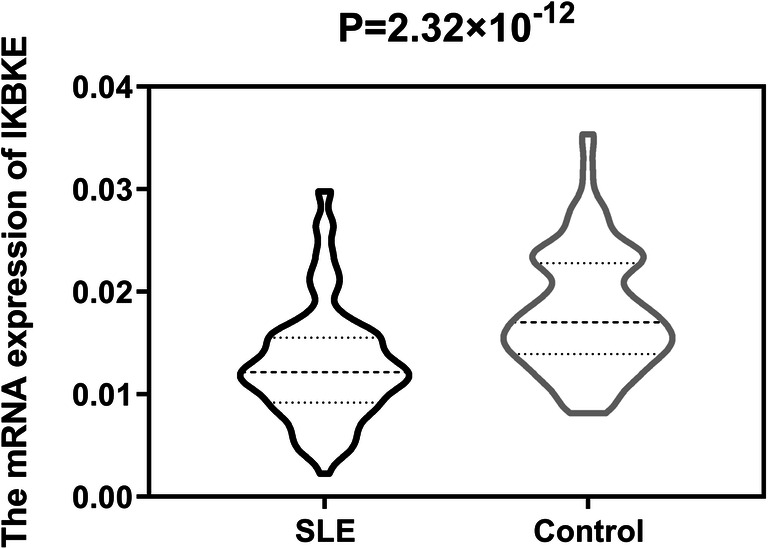
The difference of *IKBKE* mRNA expression levels between SLE patients (*n* = 121) and healthy controls (*n* = 128) in PBMCs. The expression level is lower in patients than in healthy controls (*P =* 2.32 × 10^−12^)

Excluding unqualified subjects, 121 SLE patients and 128 healthy controls were used for *IKBKE* gene expression statistics. As shown in Fig. 2, we revealed that the median expression levels of *IKBKE* mRNA in SLE patients (*Q*_50_ = 0.013 (*Q*_25_ = 0.009, *Q*_75_ = 0.015)) were lower than those in healthy controls (0.025 (0.014, 0.023)), with 1.92-fold increase in healthy controls compared with SLE patients (*P =* 2.32 × 10^−12^). To detect the effect from genotype and allele, we grouped SLE patients and healthy controls by genotype and allele, respectively, and then compared *IKBKE* mRNA expression in two groups (Supplementary Fig.1). The results were all consistent that the expression of *IKBKE* mRNA in SLE was decreased with respect to the controls. At the same time, we did not find that genotypes and alleles were related to the expression of *IKBKE* mRNA (Table 1).

### The relevance of *IKBKE* mRNA expression with clinical characteristics

**Fig. 3:**
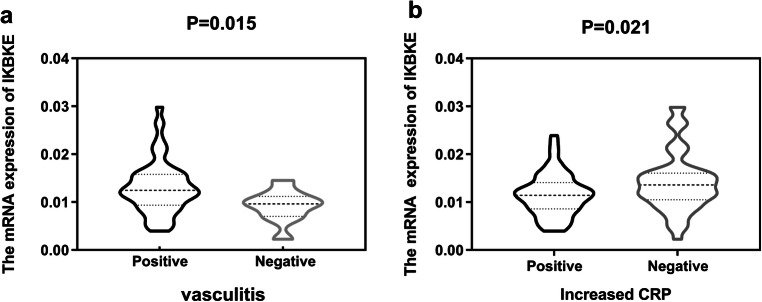
**a** The *IKBKE* mRNA expression levels were lower (*P* = 0.015) in patients with vasculitis than those without. **b** The *IKBKE* mRNA expression levels were suggestively higher (*P =* 0.021) in patients with CRP increased than those with CRP normal

In addition, we analyzed the relevance of *IKBKE* mRNA expression with clinical manifestations and laboratory indicators, respectively (Table 3). We noticed that *IKBKE* mRNA expression levels were significantly lowered (*P =* 0.015) in SLE patients with vasculitis involved than those without (Fig. 3a). And it was also suggestively lowered (*P =* 0.021) in patients with CRP increased than those with CRP normal (Fig. 3b).

**Table 3:** Relationship between *IKBKE* mRNA expression levels and SLE clinical characteristics in patients

Clinical characteristics	Presence		Absence		*P*
	N	Mean ± SD/ Q_50_(Q_25_, Q_75_)	N	Mean ± SD/ Q_50_(Q_25,_ Q_75_)	
SLEDAI ≥ 6	88	0.013 (0.009, 0.016)	33	0.012 (0.009, 0.014)	0.149
Clinical manifestations					
Renal damage	73	0.013 (0.090, 0.016)	48	0.013 (0.010, 0.014)	0.995
Malar rash	43	0.013 (0.009, 0.015)	78	0.013 (0.008, 0.020)	0.469
Photosensitivity	12	0.013 ± 0.005	109	0.013 (0.009, 0.016)	0.942
Vasculitis	11	0.009 ± 0.003	108	0.013 (0.009, 0.016)	0.015*
Hematologic involvement	68	0.012 (0.009, 0.015)	52	0.013 (0.009, 0.021)	0.829
Arthritis	20	0.015 ± 0.006	99	0.012 (0.008, 0.015)	0.208
Mucosal ulcers	13	0.016 ± 0.007	107	0.012 (0.009, 0.022)	0.071
Central nervous system involvement	5	0.011 ± 0.004	114	0.013 (0.009, 0.016)	0.450
Serositis	20	0.011 ± 0.005	100	0.014 (0.010, 0.016)	0.205
Laboratory indicators					
ANA (+)	118	0.013 (0.009, 0.016)	2	0.009 ± 0.005	0.966
Anti-dsDNA (+)	67	0.013 (0.009, 0.016)	53	0.013 (0.009, 0.015)	0.261
Anti-SM (+)	61	0.015 (0.010, 0.017)	59	0.011 ± 0.004	0.061
Increased CRP	66	0.012 (0.009, 0.014)	52	0.015 (0.009, 0.014)	0.021*
Increased ESR	94	0.013 (0.010, 0.016)	26	0.012 (0.009, 0.015)	0.438
Low complementary	79	0.014 (0.010, 0.016)	41	0.011 ± 0.004	0.071

N: number; SLEDAI: Systemic Lupus Erythematosus Disease Activity Index;

*P < 0.05;

*p* value is considered statistically significant if < 0.05

## Discussion

We had reported rs2297550 located in *IKBKE* region is significantly associated with SLE in a published GWAS [4]. Moreover, previous studies found that the SNP rs1539241, rs12142086, and rs2151222 of *IKBKE* region are associated with SLE by candidate gene study [16, 17]. However, the function of these SNPs in SLE remains to be confirmed. In this study, we predicted the regulatory effect of rs2297550 by various databases, analyzed the effect of rs2297550 on *IKBKE* mRNA expression by eQTL, and detected the relation of *IKBKE* mRNA expression to disease clinical characteristics by qRT-PCR. The results provided important clues that variant rs2297550 may be involved in SLE by regulating *IKBKE* mRNA expression levels.

By functional annotation, we knew *IKBKE* 5′ UTR variant rs2297550 has both promoter and enhancer regulatory effects on certain immune cells, kidney, and skin. The rs2297550 was found to be a putative eQTL for *IKBKE* [4]. This study also revealed the rs2297550 can regulate the *IKBKE* mRNA expression level in PBMCs and the homozygous “GG” of risk allele with lower *IKBKE* expression levels (*P* = 0.022). Nevertheless, no significant differences were found in patients. We thought that the small sample size of patients, the disease itself, and the medical treatment may hide the true effect of SNP rs2297550 on *IKBKE* expression. In addition, we queried rs2297550 in GTEx and confirmed rs2297550-G was strongly connected with decreased mRNA expression of *IKBKE* in the skin. These bioinformatic insights may further support the function of rs2297550 in the regulation of *IKBKE* mRNA expression.

*IKBKE* encodes B cell kinase ε κ light chain gene enhancer inhibitor (IKKε) which participates in multiple immune signaling pathways, such as NF-κB signaling pathway [18]. The presence of IKKε would enhance the antiapoptotic function of NF-κB in response to DNA damage [19]. In addition, the lack of IKKε may active tumor necrosis factor (TNF) signaling which is strongly linked to activation NF-κB with the result of triggering the untoward death of the cell [20, 21]. Therefore, we intend to draw a role that *IKBKE* possibly as a mediator affect NF-κB signaling pathway and protect from untoward death of the cell in SLE. All these indicated the lower expression levels of *IKBKE* may contribute to the incidence and development of SLE.

Furthermore, we found that *IKBKE* mRNA expression levels were associated with vasculitis in SLE patients (*P* = 0.015). Vasculitis is characterized by inflammatory cell infiltration and subsequent vascular wall necrosis, which is one of the most characteristic processes in the clinical evolution of SLE [22]. From the above discussion, we may think that the lack of IKKε increases the untoward death of the cell and accelerates the necrosis of the vessel wall. Some studies showed that TNF stimulates the production of CRP [23–25]. So it is reasonable to believe that the lack of IKKε promotes TNF signaling which leads to the elevation of CRP. Therefore, in this study, the patients with lower expression levels of *IKBKE* tended to display higher CRP levels (*P* = 0.021).

In summary, we dissected the contribution of the variant rs2297550 to the susceptibility of SLE and provided particularly functional hypotheses for *IKBKE* that participates in the associated signals of SLE pathogenesis. Our research may contribute to improve our knowledge about the pathogenesis of SLE and provide directions for some novel drug development and clinical interventions in SLE. However, current research has some limitations. Firstly, the sample size is limited. Besides, most patients with SLE received corticosteroids or immunosuppressive treatment. These medicines may affect our results. More extensive large-scale replication and rigorous researches are necessary in the future. Larger sample size may significantly reduce the influences from medical treatment and increase the statistical power.

## Electronic supplementary material

ESM 1 (DOCX 26 kb)

ESM 2 (PNG 442 kb)

High Resolution Image (TIF 1402 kb)
