# Supplementary material for: The decreased expression of IKBKE in systemic lupus erythematosus
Source: Clin Rheumatol. 2020 Mar 7;39(9):2611–7. doi: 10.1007/s10067-020-05006-6 (PMC7426285; doi:10.1007/s10067-020-05006-6)
Supplement: Supplementary file 1 — (DOCX 26 kb) [file 10067_2020_5006_MOESM1_ESM.docx]

|  |  | **Total *** | |  | **Chinese**  **GWAS** | |  | **Chinese Replication** | |  | **Total **** | |  | **European**  **GWAS** | |  | **European Replication** | |  | **Meta**  **ALL** | |
| --- | --- | --- | --- | --- | --- | --- | --- | --- | --- | --- | --- | --- | --- | --- | --- | --- | --- | --- | --- | --- | --- |
| **SNP** | **Gene** | **4,702 Cases** | **8,445 Controls** |  | **1,659 Cases** | |  | **3,043 Cases** | |  | **5,514 Cases** | **13,884 Controls** |  | **4,036 Cases** | |  | **1,478 Cases** | |  | **11,381 Cases** | |
|  |  |  |  |  | **3,398 Controls** | |  | **5,074 Controls** | |  |  |  |  | **6,959 Controls** | |  | **6,925Controls** | |  | **24,463 Controls** | |
|  |  | **MAF** | **MAF** |  | **OR** | ***P*** |  | **OR** | ***P*** |  | **MAF** | **MAF** |  | **OR** | ***P*** |  | **OR** | ***P*** |  | **OR** | ***P*-meta** |
| rs2297550 | *IKBKE* | 0.577 | 0.546 |  | 1.18 | 1.52×10^−4^ |  | 1.13 | 2.14×10^−4^ |  | 0.14 | 0.12 |  | 1.18 | 1.59×10^−4^ |  | NA | NA |  | 1.16 | 1.31×10^−11^ |

**Supplementary Table 1. The results of an association study on rs2297550 with SLE in Chinese and European**

*Total comprises the Chinese GWASs (1,659 cases and 3,398 controls) and the Chinese replication study (3,043 cases and 5,074 controls);

**Total comprises the European GWASs (4,036 cases and 6,959 controls) and the European replication study (1,478 cases and 6,925 controls);

ALL comprises the Chinese and the European,

MAF: minor allele frequencies (In this study, “G” is minor allele);

OR: odds ratio;

NA is placed where SNP failed QC in the Chinese data due to IMPUTE INFO scores less than 0.7.

**Supplementary Table 2. The allelic and genotypic frequencies of SNP rs2297550 in different populations**

| **Population** | **Allele**  **frequency (count)** | |  | **Genotype**  **frequency (count)** | | |
| --- | --- | --- | --- | --- | --- | --- |
|  | **G** | **C** |  | **GG** | **CC** | **GC** |
| ALL* | 0.238 (1190) | 0.762 (3818) |  | 0.084 (211) | 0.609 (1525) | 0.307 (768) |
| AFR | 0.097 (128) | 0.903 (1194) |  | 0.008 (5) | 0.814 (538) | 0.179 (118) |
| - ACB | 0.089 (17) | 0.911 (175) |  | 0.010 (1) | 0.833 (80) | 0.156 (15) |
| - ASW | 0.066 (8) | 0.934 (114) |  | / | 0.869 (53) | 0.131 (8) |
| - ESN | 0.116 (23) | 0.884 (175) |  | / | 0.768 (76) | 0.232 (23) |
| - LWK | 0.141 (28) | 0.859 (170) |  | 0.040 (4) | 0.758 (75) | 0.202 (20) |
| - MSL | 0.065 (11) | 0.935 (159) |  | / | 0.871 (74) | 0.129 (11) |
| - YRI | 0.130 (28) | 0.870 (188) |  | / | 0.741 (80) | 0.259 (28) |
| AMR | 0.303 (210) | 0.697 (484) |  | 0.107 (37) | 0.501 (174) | 0.392 (136) |
| - CLM | 0.239 (45) | 0.761 (143) |  | 0.064 (6) | 0.585 (55) | 0.351 (33) |
| - MXL | 0.273 (35) | 0.727 (93) |  | 0.047 (3) | 0.500 (32) | 0.453 (29) |
| - PEL | 0.506 (86) | 0.494 (84) |  | 0.271 (23) | 0.259 (22) | 0.471 (40) |
| - PUR | 0.212 (44) | 0.788 (164) |  | 0.048 (5) | 0.625 (65) | 0.327 (34) |
| EAS | 0.519 (523) | 0.481 (485) |  | 0.270 (136) | 0.232 (117) | 0.498 (251) |
| - CDX | 0.559 (104) | 0.441 (82) |  | 0.333 (31) | 0.215 (20) | 0.452 (42) |
| - CHB | 0.568 (117) | 0.432 (89) |  | 0.301 (31) | 0.165 (17) | 0.534 (55) |
| - CHS | 0.510 (107) | 0.490 (103) |  | 0.267 (28) | 0.248 (26) | 0.486 (51) |
| - JPT | 0.495 (103) | 0.505 (105) |  | 0.231 (24) | 0.240 (25) | 0.529 (55) |
| - KHV | 0.465 (92) | 0.535 (106) |  | 0.222 (22) | 0.293 (29) | 0.485 (48) |
| EUR | 0.113 (114) | 0.887 (892) |  | 0.016 (8) | 0.789 (397) | 0.195 (98) |
| - CEU | 0.076 (15) | 0.924 (183) |  | / | 0.848 (84) | 0.152 (15) |
| - FIN | 0.131 (26) | 0.869 (172) |  | 0.010 (1) | 0.747 (74) | 0.242 (24) |
| - GBR | 0.110 (20) | 0.890 (162) |  | 0.022 (2) | 0.802 (73) | 0.176 (16) |
| - IBS | 0.103 (22) | 0.897 (192) |  | 0.019 (2) | 0.813 (87) | 0.168 (18) |
| - TSI | 0.145 (31) | 0.855 (183) |  | 0.028 (3) | 0.738 (79) | 0.234 (25) |
| SAS | 0.220 (215) | 0.780 (763) |  | 0.051 (25) | 0.611 (299) | 0.337 (165) |
| - BEB | 0.233 (40) | 0.767 (132) |  | 0.070 (6) | 0.605 (52) | 0.326 (28) |
| - GIH | 0.189 (39) | 0.811 (167) |  | 0.029 (3) | 0.650 (67) | 0.320 (33) |
| - ITU | 0.221 (45) | 0.779 (159) |  | 0.069 (7) | 0.627 (64) | 0.304 (31) |
| - PJL | 0.177 (34) | 0.823 (158) |  | 0.010 (1) | 0.656 (63) | 0.333 (32) |
| - STU | 0.279 (57) | 0.721 (147) |  | 0.078 (8) | 0.520 (53) | 0.402 (41) |

*ALL is used this means that all individuals from that release are being considered. AFR is African; ACB is African Caribbeans in Barbados; ASW is Americans of African Ancestry in SW USA; ESN is Esan in Nigeria; LWK is Luhya in Webuye, Kenya; MSL is Mende in Sierra Leone; YRI is Yoruba in Ibadan, Nigeria; AMR is Ad Mixed American; CLM is Colombians from Medellin, Colombia; MXL is Mexican Ancestry from Los Angeles USA; PEL is Peruvians from Lima, Peru ; PUR is Puerto Ricans from Puerto Rico; EAS is East Asian; CDX is Chinese Dai in Xishuangbanna, China; CHB is Han Chinese in Beijing, China; CHS is Southern Han Chinese; JPT is Japanese in Tokyo, Japan; KHV is Kinh in Ho Chi Minh City, Vietnam; EUR is European; CEU is Utah Residents (CEPH) with Northern and Western European Ancestry; FIN is Finnish in Finland; GBR is British in England and Scotland; IBS is Iberian Population in Spain; TSI is Toscani in Italia;SAS is South Asian; BEB is Bengali from Bangladesh; GIH is Gujarati Indian from Houston, Texas; ITU is Indian Telugu from the UK; PJL is Punjabi from Lahore, Pakistan; STU is Sri Lankan Tamil from the UK.

**Supplementary Table 3.** **Association of different genotypes and alleles of rs2297550 with SLE clinical characteristics**

| **Clinical characteristics** | **GG**  **(47) *** | **GC**  **(64)** | **CC**  **(23)** | ***P*** | **G**  **(111)** | **C**  **(87)** | ***P*** |
| --- | --- | --- | --- | --- | --- | --- | --- |
| Age onset (years) | 31.39±13.03 | 27.69±10.92 | 30.43±9.77 | 0.163 | 29.31±11.96 | 28.47±10.61 | 0.287 |
| SLEDAI | 10.45 ±6.83 | 12.38±7.81 | 10.43±7.34 | 0.334 | 11.58±7.44 | 11.86±7.69 | 0.792 |
| Clinical phenotype |  |  |  |  |  |  |  |
| - Renal damage (+) | 26(55.32%) ** | 44(68.75%) | 13(56.52%) | 0.298 | 70(26.40%) | 57(44.15%) | 0.721 |
| - Malar rash (+) | 20(42.55%) | 26(40.62%) | 5(21.74%) | 0.204 | 46(41.44%) | 31(35.63%) | 0.405 |
| - Vasculitis (+) | 4(8.51%) | 5(7.81%) | 3(1.3%) | 0.666 | 9(8.11%) | 8(9.20%) | 0.786 |
| - Hematologic involvement (+) | 21(44.68%) | 32(50%) | 7(30.43%) | 0.270 | 53(47.75%) | 39(44.83%) | 0.683 |
| - Arthritis (+) | 7(14.89%) | 12(18.75%) | 3(13.04%) | 0.860 | 19(17.12%) | 15(17.24%) | 0.982 |
| - Mucosal ulcers (+) | 4(8.51%) | 9(16.36%) | 4(17.39%) | 0.540 | 13(11.71%) | 13(14.94%) | 0.504 |
| - Serositis (+) | 8(18.61%) | 10(15.63%) | 4(82.61%) | 0.980 | 18(16.22%) | 14(16.09%) | 0.959 |
| Laboratory index |  |  |  |  |  |  |  |
| - ANA (+) | 46(97.87%) | 62(96.87%) | 23(100%) | 0.990 | 108(97.30%) | 85(97.70%) | 0.857 |
| - Anti-dsDNA (+) | 20(42.55%) | 40(62.5%) | 14(60.87%) | 0.094 | 60(54.05%) | 54(62.07%) | 0.257 |
| - Anti-SM (+) | 21(44.68%) | 34(53.13%) | 11(47.83%) | 0.749 | 55(49.55%) | 45(51.72%) | 0.761 |
| - Increased CRP (+) | 27(62.79%) | 33(51.56%) | 11(47.83%) | 0.714 | 60(54.05%) | 44(50.57%) | 0.627 |
| - Increased ESR (+) | 38(88.37%) | 45(70.31%) | 18(78.26%) | 0.418 | 83(74.77%) | 63(72.41%) | 0.708 |
| - Low Complementary (+) | 30(63.83%) | 46(71.88%) | 14(60.87%) | 0.524 | 76(68.47%) | 60(68.97%) | 0.940 |

* The number of subjects with genotype "GG";

SLEDAI: Systemic Lupus Erythematosus Disease Activity Index;

** The percentage of subjects with renal damage in the genotype "GG" subjects;

*P* value is considered statistically significant if < 0.05.

**Supplementary Fig.1.** In groups of different genotypes ((**a.** "GG"), (**b**. "GC") and (**c**. "CC")) and alleles ((**d**. "G") and (**e.** "C")) , the *IKBKE* mRNA expression levels are all lower in SLE patients than in healthy controls (*P*=0.002, *P*<0.0001, *P*<0.0001, *P*<0.0001, *P*<0.0001, respectively).
