# Supplementary figures and images for: The decreased expression of IKBKE in systemic lupus erythematosus
Source: Clin Rheumatol. 2020 Mar 7;39(9):2611–7. doi: 10.1007/s10067-020-05006-6 (PMC7426285; doi:10.1007/s10067-020-05006-6)

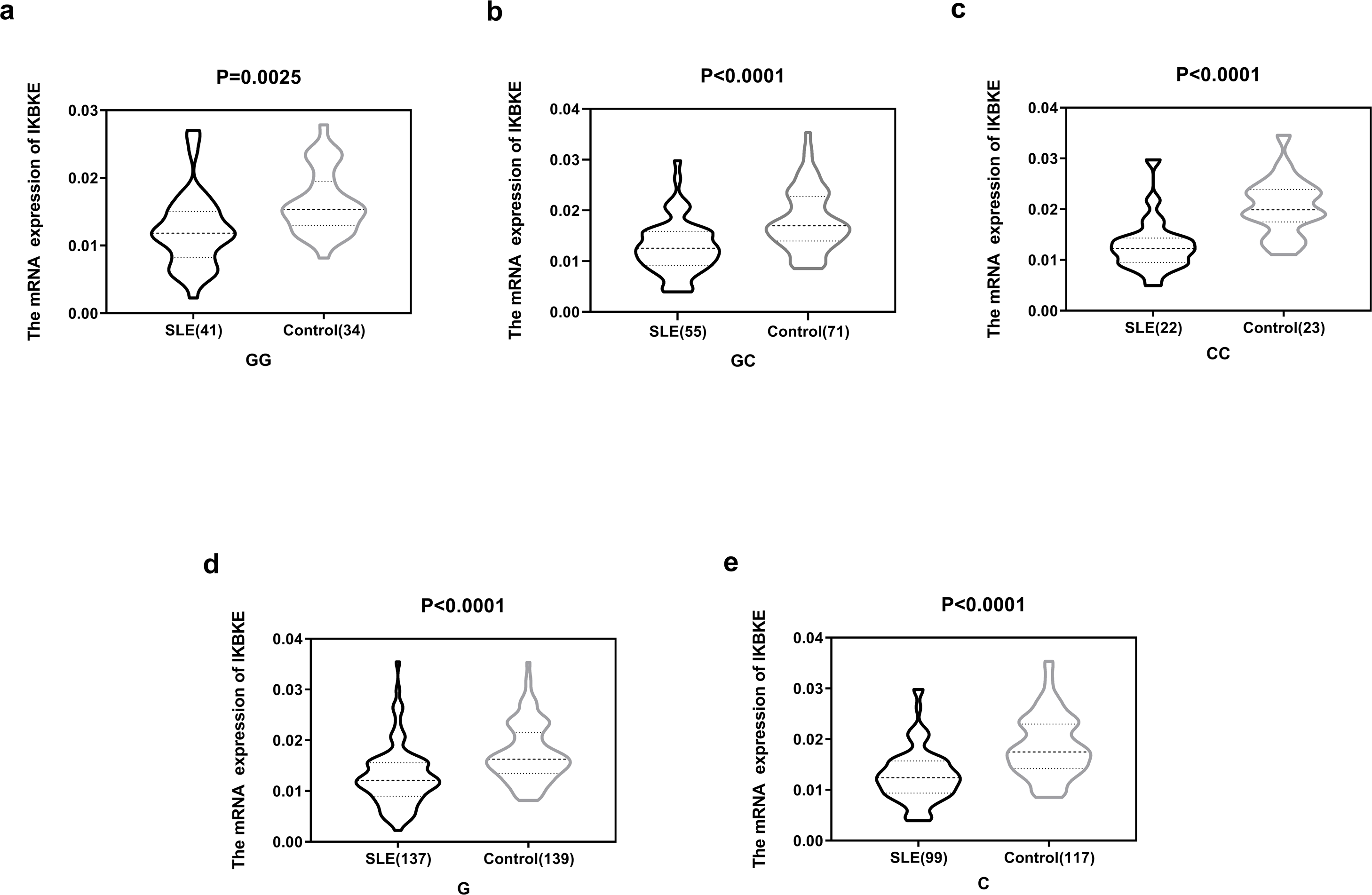

Supplement: Supplementary file 2 — (PNG 442 kb) [file 10067_2020_5006_Fig4_ESM.png]

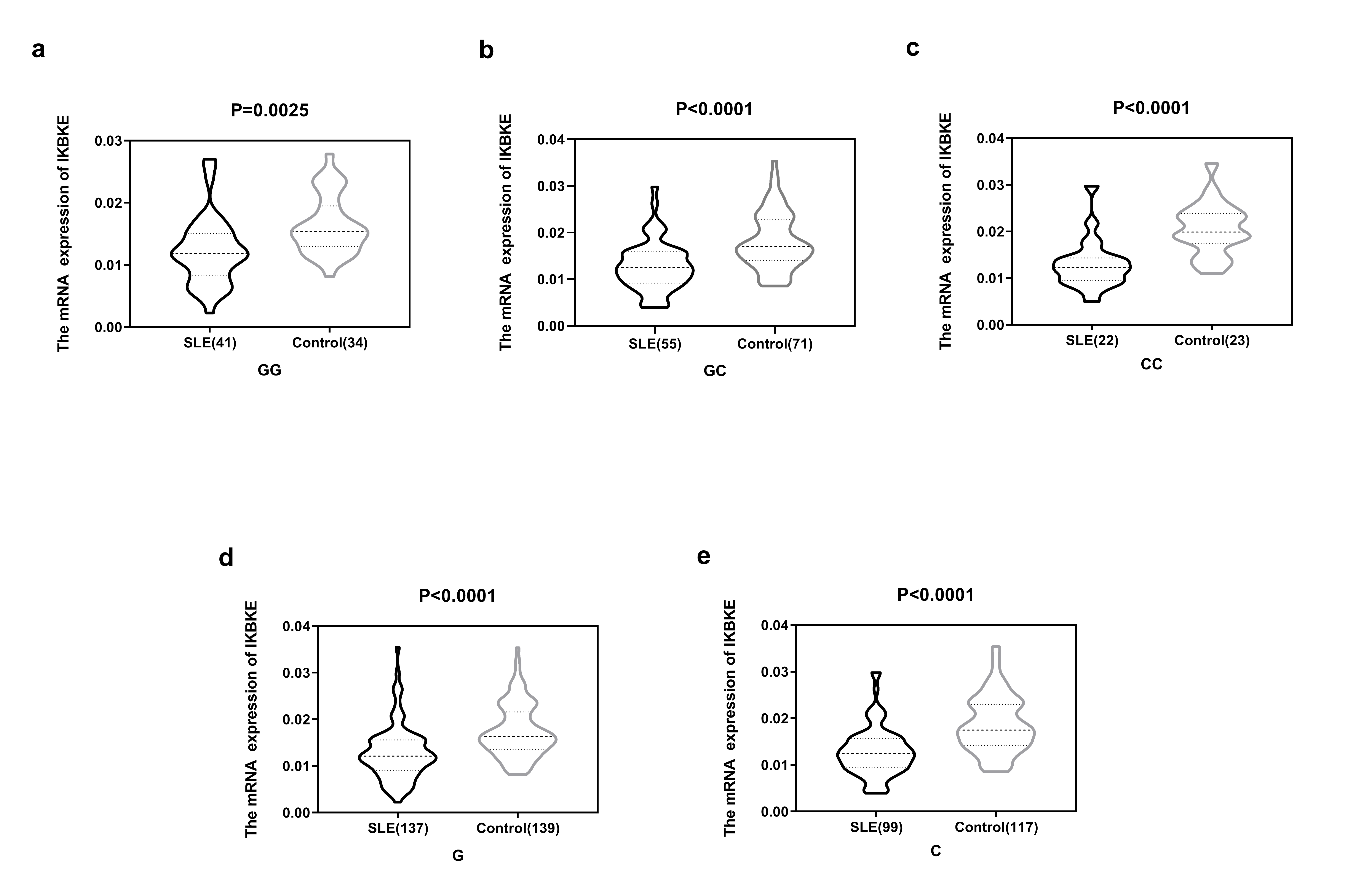

Supplement: Supplementary file 3 — High Resolution Image (TIF 1402 kb) [file 10067_2020_5006_MOESM2_ESM.tif]
